# YAP promotes neural crest emigration through interactions with BMP and Wnt activities

**DOI:** 10.1186/s12964-019-0383-x

**Published:** 2019-06-22

**Authors:** Deepak Kumar, Erez Nitzan, Chaya Kalcheim

**Affiliations:** Department of Medical Neurobiology, IMRIC and ELSC, Hebrew University of Jerusalem-Hadassah Medical School, P.O. Box 12272, 91120 Jerusalem, Israel

**Keywords:** Apoptosis, Avian embryo, Cell delamination, Cell proliferation, Epithelial-to-mesenchymal transition, FoxD3, Somite

## Abstract

**Background:**

Premigratory neural crest progenitors undergo an epithelial-to-mesenchymal transition and leave the neural tube as motile cells. Previously, we showed that BMP generates trunk neural crest emigration through canonical Wnt signaling which in turn stimulates G1/S transition. The molecular network underlying this process is, however, not yet completely deciphered.

Yes-associated-protein (YAP), an effector of the Hippo pathway, controls various aspects of development including cell proliferation, migration, survival and differentiation. In this study, we examined the possible involvement of YAP in neural crest emigration and its relationship with BMP and Wnt.

**Methods:**

We implemented avian embryos in which levels of YAP gene activity were either reduced or upregulated by in ovo plasmid electroporation, and monitored effects on neural crest emigration, survival and proliferation. Neural crest-derived sensory neuron and melanocyte development were assessed upon gain of YAP function. Imunohistochemistry was used to assess YAP expression. In addition, the activity of specific signaling pathways including YAP, BMP and Wnt was monitored with specific reporters.

**Results:**

We find that the Hippo pathway transcriptional co-activator YAP is expressed and is active in premigratory crest of avian embryos. Gain of YAP function stimulates neural crest emigration in vivo, and attenuating YAP inhibits cell exit. This is associated with an accumulation of *FoxD3*-expressing cells in the dorsal neural tube, with reduced proliferation, and enhanced apoptosis. Furthermore, gain of YAP function inhibits differentiation of Islet-1-positive sensory neurons and augments the number of *EdnrB2*-positive melanocytes. Using specific in vivo reporters, we show that loss of YAP function in the dorsal neural tube inhibits BMP and Wnt activities whereas gain of YAP function stimulates these pathways. Reciprocally, inhibition of BMP and Wnt signaling by noggin or Xdd1, respectively, downregulates YAP activity. In addition, YAP-dependent stimulation of neural crest emigration is compromised upon inhibition of either BMP or Wnt activities. Together, our results suggest a positive bidirectional cross talk between these pathways.

**Conclusions:**

Our data show that YAP is necessary for emigration of neural crest progenitors. In addition, they incorporate YAP signaling into a BMP/Wnt-dependent molecular network responsible for emigration of trunk-level neural crest.

## Background

The neural crest (NC), a transient embryonic population of progenitors, leads to a remarkable range of adult derivatives [1, 2]. It not only generates a significant fraction of the peripheral nervous system including sensory, sympathetic, parasympathetic and enteric neurons as well as all the peripheral glia including Schwann cells, but also distinct non neural cell types such as melanocytes, endocrine cells and various types of mesenchymal cells that compose the craniofacial skeleton [1, 3–5].

NC cells are epithelial in the dorsal neural tube (NT) and then undergo an epithelial-to-mesenchymal transition (EMT) in a spatiotemporally regulated manner [6]. Disassembly of cell-cell junctions alongside the loss of apico-basal polarity are prerequisites for NC EMT and emigration. Concomitantly, cells degrade the overlying basement membrane in order to invade the extracellular matrix, processes considered to be essential for the acquisition of cell motility [7]. Core EMT triggers, effectors and modulators including secreted morphogens, transcription factors, cell adhesion molecules, RhoGTPases, etc., have been uncovered [6, 8–11]. Moreover, regulation of their activity occurs at transcriptional, translational and epigenetic levels [7, 12–14].

We previously showed that a balance between BMP and its inhibitor noggin, in association with the developing somites, underlies the emigration of trunk-level NC [15–17]. BMP induces EMT of NC by triggering *Wnt1* transcription that in turn promotes G1/S transition, a necessary step for delamination of trunk NC [18, 19]. N-cadherin and Rho/Rac GTPases are also part of the BMP-dependent network of genes with activity on NC emigration [10, 20]. Subsequently, it was found that dynamic counter-gradients of FGF8 and retinoic acid in the paraxial mesoderm affect NC EMT partly through the modulation of specific aspects of BMP and Wnt signaling [21]. Being such an essential and multifaceted process, it expected that the regulation of NC EMT is highly complex.

The tumor suppressor Hippo/MST1/2 is an evolutionary conserved pathway that controls various aspects of development including cell proliferation, migration, survival and differentiation as well as adult homeostasis and tumorigenesis [22–24]. Yes-associated-protein (YAP) and the transcriptional co-activator with PDZ-binding-motif (TAZ) are the major effectors of Hippo signaling [25]. YAP and TAZ associate with DNA-binding transcription factors, such as TEAD1–4, to regulate downstream gene expression [24, 26, 27]. Upstream Hippo/MST kinase cascades phosphorylate and inactivate TAZ/YAP, thereby preventing their nuclear translocation and leading to their ubiquitin-mediated degradation; when the Hippo kinase is inactive, YAP translocates into the nucleus where it exerts its transcriptional activity [28–31]. In turn, the activity of TAZ/YAP is tightly regulated in response to specific molecular and mechanical signals emanating from the microenvironment [22, 23, 32–37].

A few studies addressed the roles of YAP on NC derivatives. YAP signaling is involved in the expansion of dorsal root ganglion (DRG) progenitors and glia, temporary inhibition of sensory neuron formation [38], expression of the microphtalmia gene in melanocytes [39], formation of Schwann cells and their myelination [40], and in aspects of craniofacial and smooth muscle ontogeny [34, 41, 42]. In contrast, with the exception of the findings that YAP is necessary for generation of cranial NC in zebrafish [43] and of Pax3 expression in frog NC [44], little is known about its function during early NC development.

In the present study, we show that YAP immunoreactive protein is expressed in the dorsal NT of avian embryos and in early migrating NC cells, but not in coalescing peripheral ganglia. Consistently, implementing a specific YAP/TEAD reporter confirms activity in the premigratory and early migrating NC. Gain of YAP function stimulates NC emigration; in contrast, attenuation of YAP inhibits the exit of NC cells while reducing cell proliferation and survival. Being unable to leave the NT, *FoxD3*-expressing cells accumulate in the dorsal NT. Using specific BMP and Wnt reporters, we show that loss of YAP function in the dorsal NT inhibits the activity of these pathways. Conversely, inhibition of BMP or Wnt signaling reduces YAP activity and YAP-mediated NC emigration. In addition, in avian embryos, YAP overexpression inhibits neuronal differentiation and enhances the number of *Ednrb2*-expressing melanocytes. Together, the present data expand our knowledge on the mechanisms of NC EMT by integrating YAP function within a BMP/Wnt molecular network.

## Materials and methods

### Avian embryos

Fertile quail (*Coturnix coturnix japonica*) and chick (*Gallus gallus*) eggs were from commercial sources (Moshav Mata).

### Expression vectors and electroporation

pCAGGS-AFP [45] was used as control. The following expression vectors of chick origin were generously provided by Xinwei Cao with permission of Sam Pfaff and Fred Gage [46]: full length YAP, a kinase dead version of Hippo/Mst2 (Mst2^KD^) that has a dominant-negative effect on the endogenous kinase [47], a truncation mutant (YAP^279^) lacking the activation domain; this construct remains cytoplasmic upon transfection and therefore serves as control; and YAP_N_^279^ with a nuclear localization signal (YAP-NLS), shown to abrogate YAP activity [46]. In addition, a shRNA to YAP (shYAP #1) and a control shRNA consisting of a scrambled sequence of shYAP. To abrogate BMP and Wnt activities, plasmids encoding the BMP inhibitor noggin and a truncated form of Xdishevelled (pCAGGS-Xdd1-YFP) were implemented, respectively [18].

For electroporations, DNA (1–4 μg/μl) was microinjected into the lumen of the NT of 15–18- somite-stage (ss) embryos at the level of the segmental plate and the two recently formed somites. For hemi NT electroporations, 5 mm tungsten electrodes were placed on either side of the embryo. For discrete electroporations into the dorsal NT, a 5 mm tungsten electrode was inserted under the blastoderm and a fine, 1–2 mm long electrode placed over the dorsal NT [48]. A square wave electroporator (BTX, San Diego, CA) was used to deliver 3 sequential pulses of 12 msec each. Some electroporated embryos received a pulse of Brdu (10 mM) for 1 h prior to fixation.

### In vivo reporters

The following plasmids were implemented: a genetic reporter for BMP activity consisting of a BMP responsive element (BRE) which drives expression of GFP. BRE-GFP contains two copies of two distinct and conserved elements of the binding sites for Smad4 upstream of a minimal tk promoter [49, 50]. Specificity of the BMP reporter was previously verified by co-electroporation with pEFBOS-mBMP4 [51]. A reporter containing 12 TCF binding sites upstream of a minimal promoter driving destabilized enhanced GFP (12 TOPFLASH-d2EGFP) was used to detect Wnt β-catenin dependent activity [52]. In addition, to determine the presence of YAP-TEAD activity [53, 54], we implemented the 8xGTIIC-luciferase reporter (plasmid: 34615; Addgene, Cambridge, MA) followed by immunostaining with a rabbit polyclonal anti-luciferase antibody (Abcam, ab21176) and fluorescent detection with a RFP-anti rabbit antibody (code #111–295-144; Jackson Immunoresearch, PA).

### Tissue processing, immunohistochemistry and in situ hybridization

Avian embryos were fixed with 4% formaldehyde, embedded in paraffin wax and sectioned at 5 or 8 μm. Immunostaining for GFP (Molecular Probes), Islet-1, and Brdu (all from DSHB) was as described [55]. HNK-1 (mouse anti-human CD57) was from BD Biosciences. Anti-Histone H3 (phospho S10) was from Abcam (mAbcam 14,955). Nuclei were visualized with Hoechst.

For YAP immunostaining, a rabbit polyclonal (Santa Cruz SC-15407, YAP H-125) against aa206–330 of the human YAP and mouse anti-YAP [Santa Cruz SC-101199; YAP (63.7)] were used and yielded similar results. Detection of DNA fragmentation was done by TUNEL (ab66110, Abcam) according to manufacturer’s instructions.

In situ hybridization for *Foxd3* [17] and *EdnrB2* [56] was performed as described [57].

Sections were photographed using a DP73 (Olympus) cooled CCD digital camera mounted on a BX51 microscope (Olympus). For figure preparation, images were exported into Photoshop CS2 (Adobe). If necessary, the brightness and contrast were adjusted to the entire image. In all transverse sections presented, lateral is to the left and dorsal is top.

### Data analysis and statistics

The number of labeled emigrating NC cells, of Islet1+ neurons in DRG, of *EdnrB2*+ cells in the lateral pathway, of phospho-histone H3, Brdu or Tunel-positive nuclei in dorsal NT was monitored in 15–20 consecutive sections per embryo in a total of 4–10 embryos per treatment. The dorsal 20% of the NT, shown to harbor the premigratory NC at the stages examined [58], was considered for measurements of cell proliferation and apoptosis.

Results are expressed as absolute number of cells per section±SEM or as % of total transfected cells±SEM. The intensity of cells expressing the YAP reporter in an equivalent electroporated area of treated vs. control embryos was measured using Image J software (NIH) and normalized to the non-electroporated side. Significance of results was determined using the two-tailed Student’s test. A *p*-value of 5% or less was considered statistically significant.

## Results

### YAP protein is localized and active in the dorsal neural tube

**Fig. 1:**
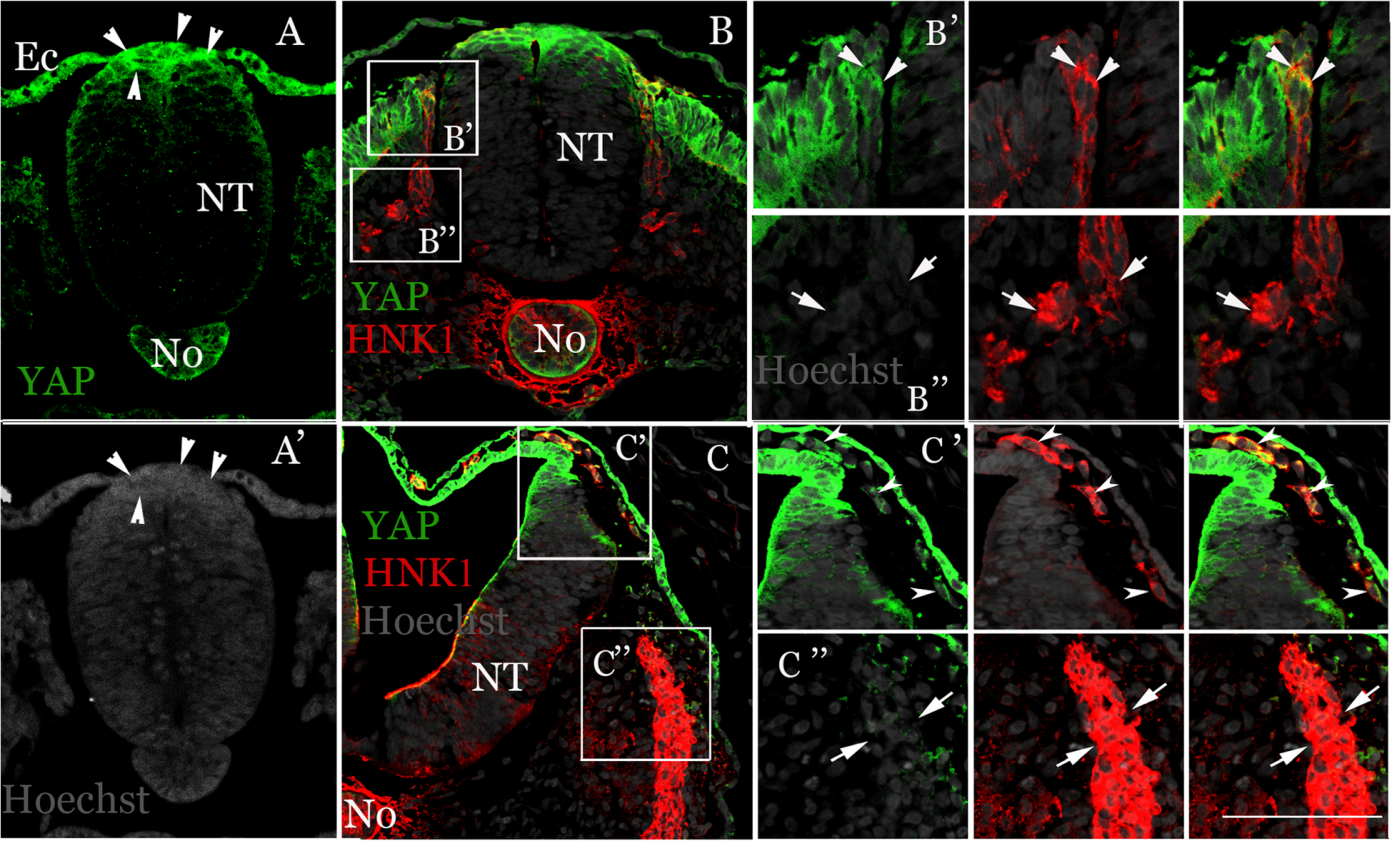
Expression of YAP-immunoreactive protein in premigratory and emigrating NC of avian embryos. Transverse sections at (**a**,**a’**) early epithelial somite level showing YAP expression in cytoplasm and nuclei (arrowheads) of cells at the premigratory domain of the dorsal neural tube (NT). Ectoderm (Ec) and notochord (No) are also positive. (**b-b**″) Dissociated somite level with migratory NC. Note in **b**,**b′** co-expression of YAP (green) and HNK-1 (red) in emigrating progenitors adjacent to the NT (arrowheads in **b**′). In contrast, cells migrating ventrally through sclerotome are HNK-1+/YAP-negative (**b**″, arrows). The dorsal NT remains positive and also the dermomyotome (DM). (**c-c**″) Midbrain level showing positive YAP signal in dorsal NT, YAP+/HNK-1+ NC cells between NT and ectodem (arrowheads in **c**′), but HNK-1+/YAP-negative in nascent ganglion (arrows in **c″**). Nuclei were stained with Hoechst. Bar = (**a**-**c**), 50 μM

Immunohistochemical detection of YAP was performed in embryos aged 18 to 25 somite pairs. In the trunk, YAP-positive cells were apparent in both the cytoplasm and nuclei of dorsal NT cells at all stages, corresponding to premigratory NC (Fig. 1a, b). Co-staining with the NC marker HNK-1 revealed that only early emigrating/migrating NC progenitors expressed YAP immunoreactive protein (Fig. 1b, b’, arrowheads) whereas cells migrating further ventral through the sclerotome were negative (Fig. 1b,b”, arrows). Likewise, at the level of the hindbrain, the thin dorsal NT and more ventral neuroepithelium expressed YAP and so were HNK-1+ cells located between ectoderm and NT (Fig. 1c,c’, arrowheads). In contrast, the nascent HNK-1+ sensory ganglion was YAP-negative (Fig. 1c, c”).

**Fig. 2:**
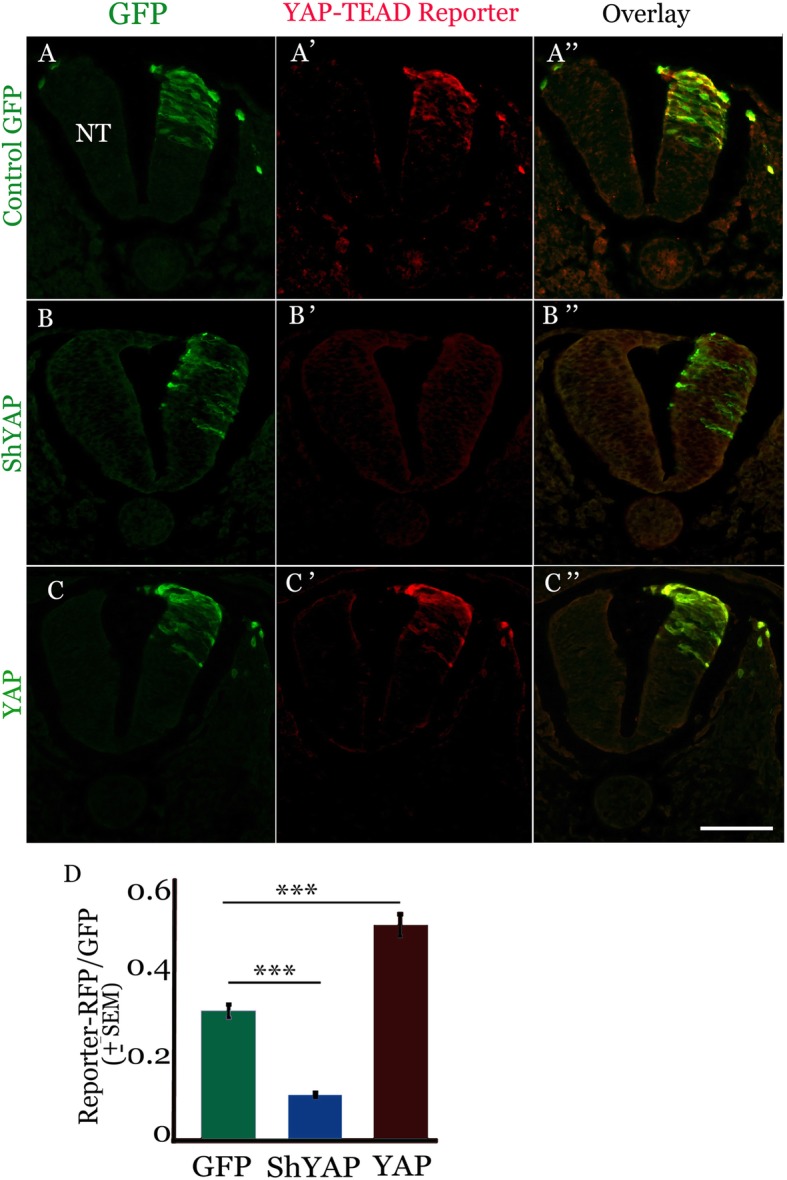
YAP is active in premigratory NC. YAP activity in neural tube (NT) and emigrating NC revealed by expression of the 8xGTIIC-luciferase reporter and fluorescent detection with RFP in control (**a-a”**), shYAP (**b-b″**), or YAP overexpression (**c-c**″). (**d**) Quantification of reporter activity. ***p < 0.0001. Bar = 50 μM

Next, we monitored YAP activity. To this end, we co-electroporated the 8xGTIIC YAP-TEAD reporter [53, 54] along with control GFP into embryos aged of 15–18 somite pairs and reincubated them for 24 h. Consistent with the immunohistochemical results, YAP activity was evident in transfected dorsal NT cells (*N* = 8, Fig. 2a-a”, d).

To control for specificity of the reporter, similar embryos were electroporated with a shRNA to YAP to attenuate its activity. This shYAP construct was previously reported to inhibit *YAP* mRNA expression and function on cell survival in NT [46]. shYAP significantly reduced YAP-TEAD reporter activity in the dorsal NT (Fig. 2b-b”, d, *N* = 5, *p* < 0.0001). Reciprocally, a YAP-encoding DNA was delivered which enhanced reporter activity in all embryos examined when compared to controls (Fig. 2c-c”, d, N = 5, *p* < 0.0001). Thus, Yap is both expressed and active in premigratory NC cells.

### YAP is necessary and sufficient for NC emigration from the dorsal NT primordium

**Fig. 3:**
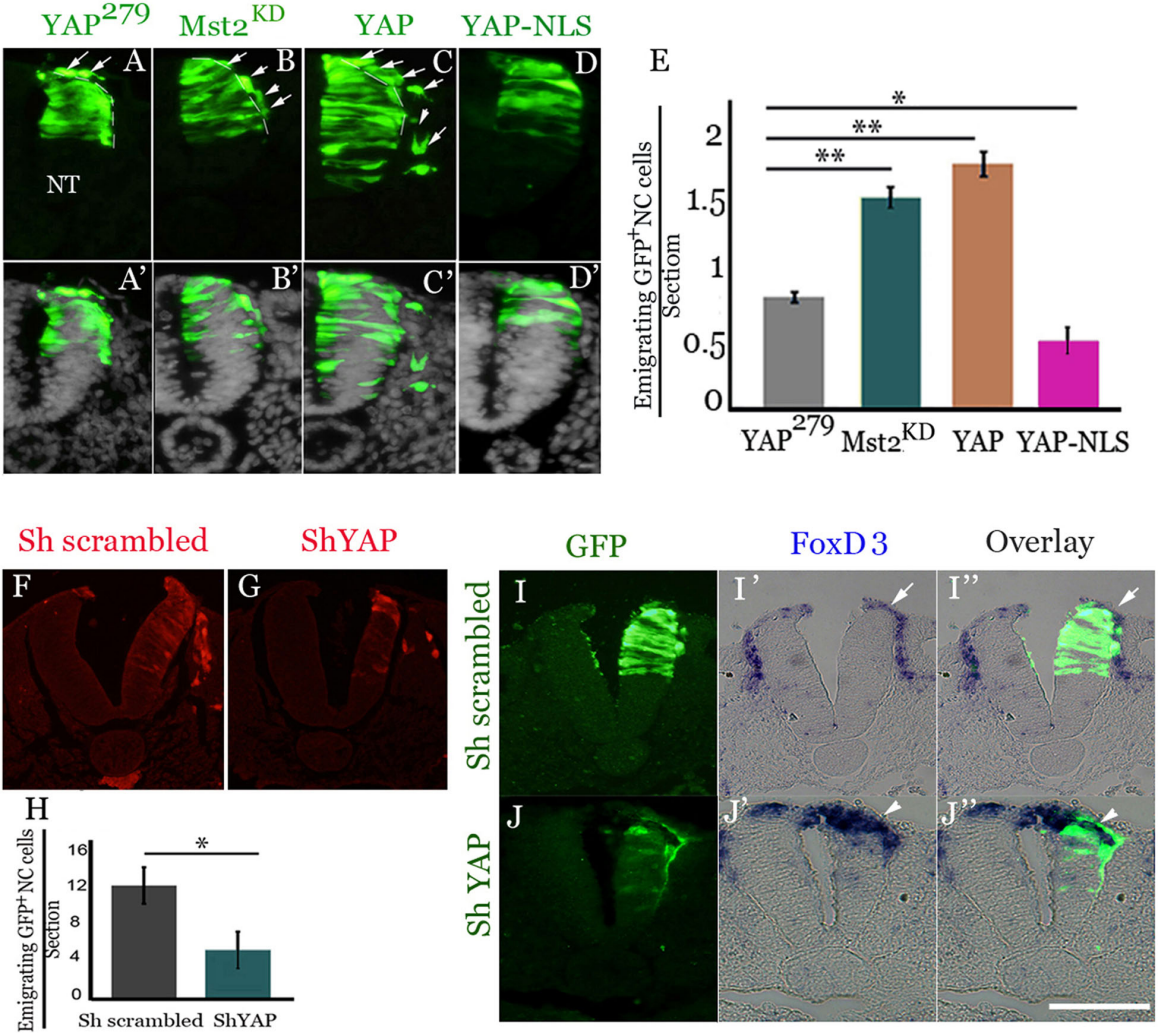
Gain or loss of YAP function, respectively, promotes or inhibits NC emigration. (**a**-**d**’) Electroporation for 16 h of control YAP^279^ (**a**,**a**’), Mst2^KD^ (**b**,**b**′), or YAP (**c**,**c**′). Mst2^KD^ and YAP activate the pathway; and YAP-NLS (**d**, **d**’) inhibits activity. Arrows depict emigrating NC cells. (**e**) Data quantification. **p < 0.0002 and **p < 0.0006 for Mst2^KD^ and YAP; *p < 0.001 for YAP-NLS. (**f**, **g**) Inhibition of YAP activity by shYAP (**g**) vs. scrambled RNA (**f**) for 24 h. (**h**) Data quantification, * *p* < 0.001. (**i**-**j**”) Accumulation of *FoxD3*+ cells in the dorsal neural tube (NT) of embryos electroporated with shYAP (**j**-**j**”, arrowheads) compared to scrambled RNA (I-I″, arrows depict *FoxD3*+ emigrated NC). Bar = (**a**-**d**’, **f**, **g**) 55 μM; (**i**-**j**”) 50 μM

A possible effect of Hippo/YAP signaling on initiation of NC emigration was examined by gain and loss of function approaches. Fifteen somite-stage (ss) embryos were electroporated with a kinase-dead version of Hippo/Mst2 (Mst2^KD^) that augments endogenous YAP signaling or with a plasmid encoding YAP [46]. Both genes significantly enhanced the number of emigrating NC cells monitored 16 h following transfection when compared to YAP^279^. Although YAP^279^ is a truncation mutant lacking the activation domain, this construct was previously demonstrated to remain cytoplasmic upon transfection [46]6], and therefore serves as control (Fig. 3a-c’, e, *N* = 6 embryos counted for each treatment, *p* < 0.0002 and *p* < 0.0006 for Hippo/Mst2^KD^ and YAP compared to control, respectively). In contrast, a reduced amount of emigrating progenitors was measured when electroporating YAP^279^ to which a nuclear localization signal was attached (YAP-NLS), that was previously shown to inhibit YAP activity [46], (Fig. 3d,d’, e, N = 6, *p* < 0.001).

To abrogate YAP by independent means, we applied a shYAP construct (Fig. 2) and compared it with a scrambled shRNA. Fifteen-18ss embryos were electroporated and reincubated for 24 h, a stage corresponding to advanced NC migration. shYAP caused a three-fold reduction in the number of migratory cells compared with the control RNA (Fig. 3f-h, *N* = 8 for each treatment, *p* < 0.001). Consistent with reduced cell emigration, an accumulation of *Foxd3*+ progenitors co-expressing shRNA and GFP was observed in the premigratory domain of the NT (Fig. 3j-j” arrowheads), when compared to the scrambled RNA controls in which *Foxd3*+ cells already left the NT by this stage (Fig. 3i-i” arrows).

These results show that YAP activity is necessary and sufficient for NC emigration.

### YAP signaling regulates proliferation and survival of premigratory NC

**Fig. 4:**
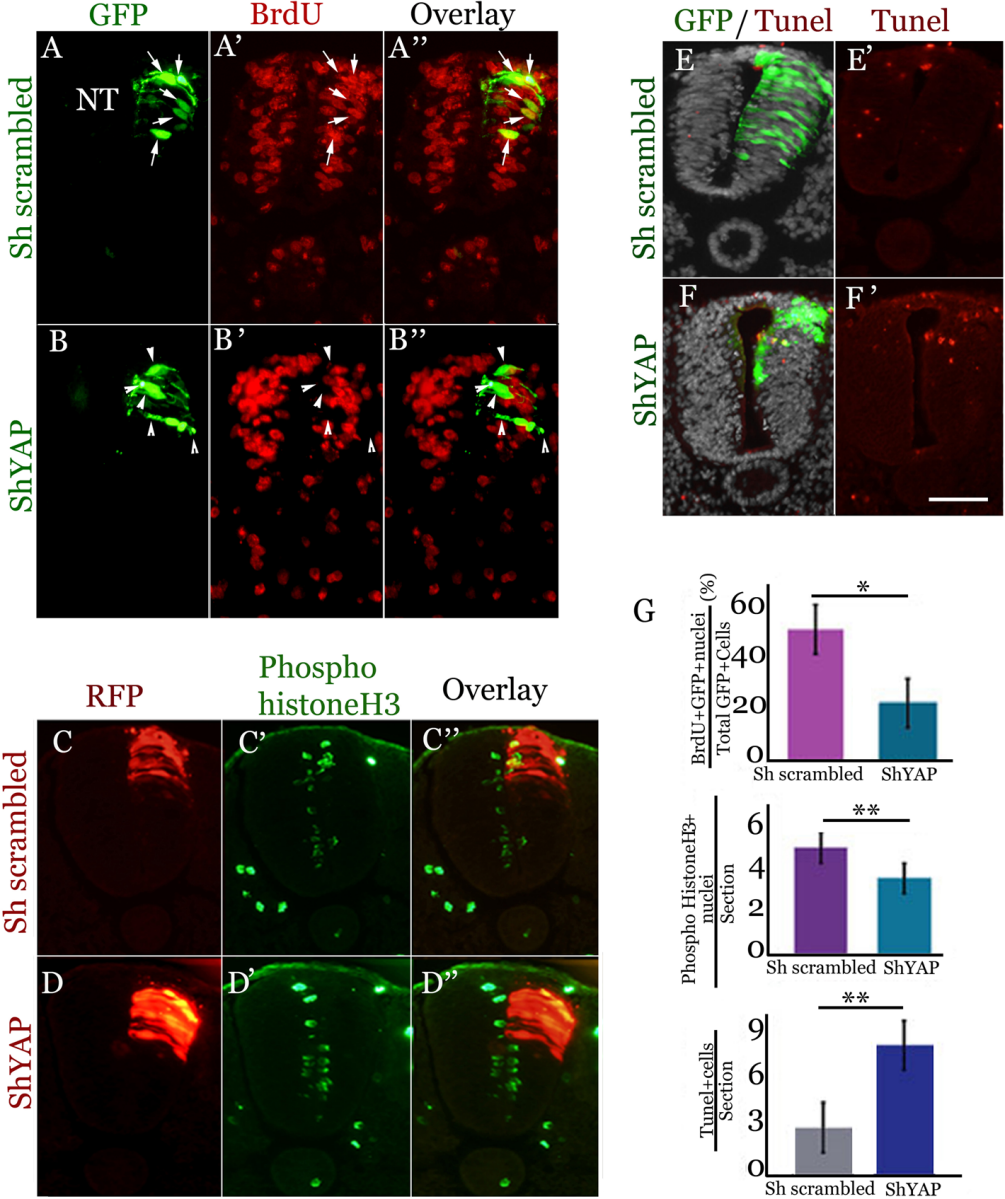
YAP stimulates G1/S transition, mitosis and survival of premigratory NC cells. (**a**-**b**″) Brdu immunostaining of S-phase nuclei (red) of scrambled RNA (**a**-**a**”) and shYAP (**b**′-**b**″)-electroporated embryos (green). Arrows in **a**-**a**” depict GFP+/Brdu+ nuclei and arrowheads in **b**-**b**″ point to GFP+/Brdu-negative cells. (**c**-**d**”) Immunostaining for mitotic figures with a phospho-histone H3 antibody (green) of scrambled RNA (**c**-**c**″) and shYAP (**d**-**d**”)-electroporated embryos (red). (**e**-**f**′) Visualization of apoptotic nuclei by Tunel (red) in scrambled RNA (**e**,**e**’) and shYAP electroporated embryos (**f**,**f**′) (green). Nuclei are visualized with Hoechst. (**g**) Data quantification restricted to electroporated cells in the dorsal 20% of the neural tube (NT). Upper graph represents incorporation of Brdu into nuclei (*p < 0.001); middle graph is the quantification of mitotic figures (**p < 0.003), and lower panel represents apoptotic nuclei (**p < 0.0007). Bar = 50 μM

We previously reported that G1/S transition is a necessary event for trunk NC cell delamination [19]. Furthermore, YAP was found to act as a mitogen in the NT [46] and in additional systems (see Introduction) and is also a necessary factor for NC emigration (Fig. 3). Thus, we examined whether loss of YAP function affects distinct phases of the NC cell cycle. Electroporation of shYAP to the dorsal NT inhibited G1/S transition (Brdu incorporation) in the transfected cells by 16 h, when compared to scrambled RNA (Fig. 4, a-b″, g, *N* = 6 for both shYAP and scrambled RNA, *p* < 0.001). shYAP also reduced the number of phospho-histone H3+ mitotic cells 24 h post-transfection (Fig. 4c-d”, g, *N* = 8 for both shYAP and scrambled RNA, *p* < 0.003).

Next, we assessed a possible effect of YAP activity on cell survival by measuring the number of Tunel+ nuclei in the dorsal 20% of ShYAP vs. scrambled RNA-treated NTs. The former caused a 2.8-fold increase in apoptotic nuclei (Fig. 4e-f’, g, *N* = 5 for both treated and controls, *p* < 0.0007).

Together, these results further strengthen the relationship between cell proliferation of premigratory NC and subsequent cell emigration and place YAP signaling as a participant in both processes. In addition, YAP may act as a survival factor for premigratory NC. Alternatively, cells that are unable to proliferate and emigrate upon attenuation of YAP activity eventually undergo apoptosis.

### YAP overexpression inhibits neuronal differentiation in avian DRG and enhances the number of *EdnrB2*-positive melanocytes

**Fig. 5:**
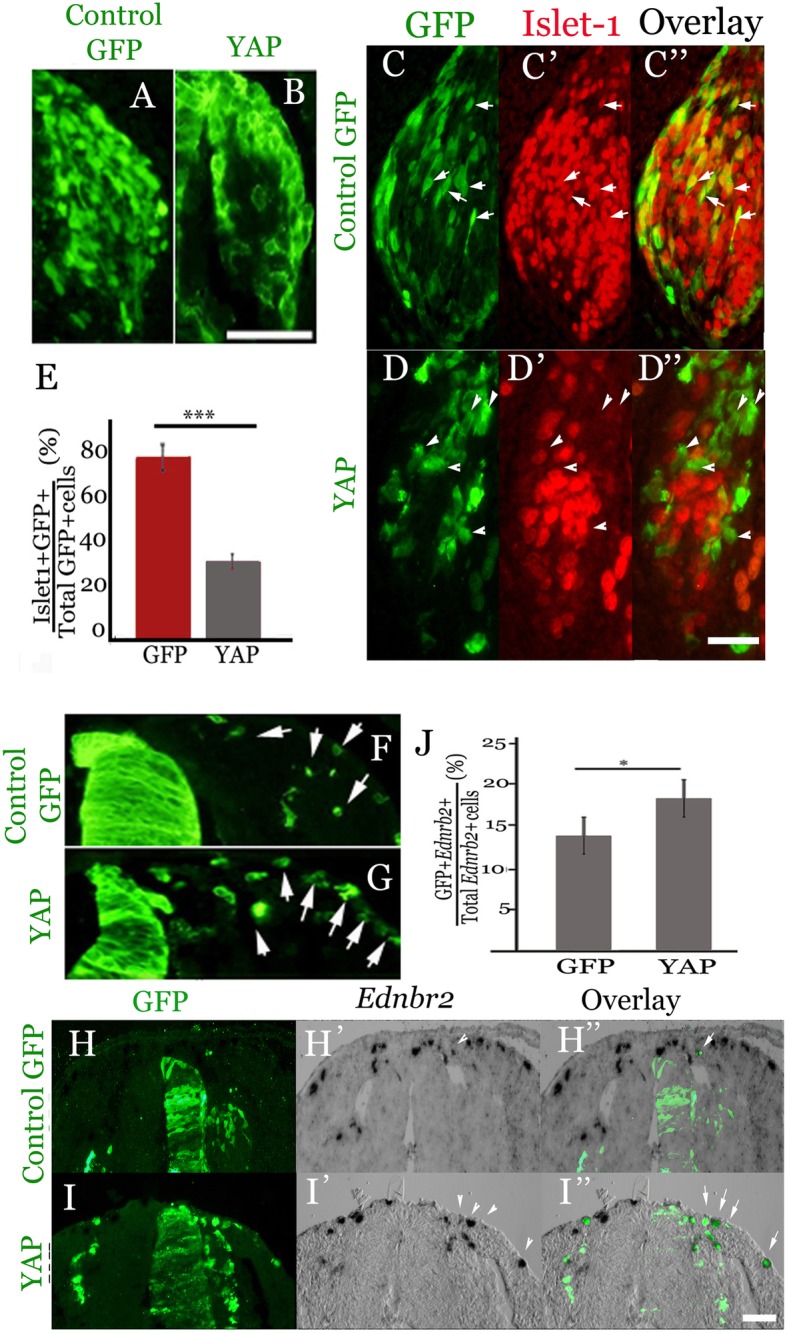
Gain of YAP function inhibits neuronal differentiation in DRG and stimulates the proportion of EdnrB2-positive melanocytes in the subectodermal pathway. (**a**-**d**”) Transverse sections of DRG in 4 day-old embryos electroporated two days earlier with control GFP (**a**, **c**-**c**″) or with YAP (**d**-**d**”). Note the even distribution of transfected cells throughout the DRG in A and its preferential peripheral distribution in B. (**c**-**d**”) Co-labeling of GFP and Islet-1. Arrows in **c**-**c**″ depict electroporated cells co-expressing GFP and Islet-1; arrowheads in D-D” point to YAP-GFP+/Islet-1-negative progenitors. (**e**) Quantification of the percentage of Islet+/GFP+ neurons over total GFP+ cells (***p < 0.0001). (**f**-**i**″) Transverse sections of 4 day-old embryos electroporated two days earlier with control GFP (**f**, **h**-**h**″) or with YAP (**g**, **i**-**i**″). (**h**-**i**″) represent co-labeling of GFP with in situ hybridization for *Ednrb2.* Arrows depict electroporated cells co-expressing GFP (green) and *Ednrb2* (blue). (**j**) Quantification of the percentage of GFP+/*Ednrb2+* melanocytes in the lateral pathway over total *Ednrb2+* cells (*p < 0.001). Bars = 50 μM

Since gain of YAP function stimulated NC delamination (Fig. 3), we examined how does this early event affect later development of NC derivatives. Electroporation of hemi-NTs with control GFP at 15-18ss resulted two days later in a rather even distribution of transfected cells throughout the developing DRG. In contrast, YAP-overexpressing cells were preferentially localized to the periphery of the ganglia that mainly harbors progenitor cells (Fig. 5 a, b), suggesting that neuronal differentiation is negatively affected. To characterize this effect, neurons were immunostained for Islet1. A significant reduction in the percentage of Islet+/GFP+ neurons over the total GFP+ cells was monitored in YAP-treated vs. control DRG (Fig. 5c-e, *N* = 5 embryos per treatment, *p* < 0.0001). These data confirm and further extend previous results obtained in mice reporting that YAP overexpression stimulated glial development and delayed formation of early differentiating, TrkC-expressing neurons [38], and suggest that YAP downregulation is necessary for subsequent neuronal differentiation.

Melanocytes are the latest population of emigrating NC progenitors in the trunk; their migration along the subectodermal pathway is driven by the EdnrB2 receptor [56, 58–61]. A previous study in mice showed that the microphtalmia (*Mitf*) gene is coordinately regulated by Pax3 and YAP/TAZ. YAP/TAZ null embryos abrogated *Mitf* expression in cells migrating along the subectodermal pathway [39], suggesting a role for YAP in aspects of melanocyte development.

Since loss of YAP function in avian embryos affected NC emigration, analysis of melanocytes as well as of other NC derivatives in such embryos was not possible. Therefore, we adopted a gain of function approach. Electroporation of YAP into the young NT resulted in an apparent increase in laterally migrating GFP+ cells (Fig. 5f, g, arrows). When combined with in situ hybridization for *EdnrB2*, a 75% increase in the percentage of *EdnrB2*+/GFP+ cells over total *EdnrB2*+ melanoblasts was monitored migrating along the dorsolateral route compared to control GFP+ cells (Fig. 5h-j, *N* = 4 embryos counted for each treatment, *p* < 0.001). This effect can be accounted for either by a direct effect of YAP on proliferation/survival of melanoblasts or by earlier effects on the proliferation, survival and/or emigration of premigratory NC cells.

### YAP signaling regulates BMP and Wnt activities

**Fig. 6:**
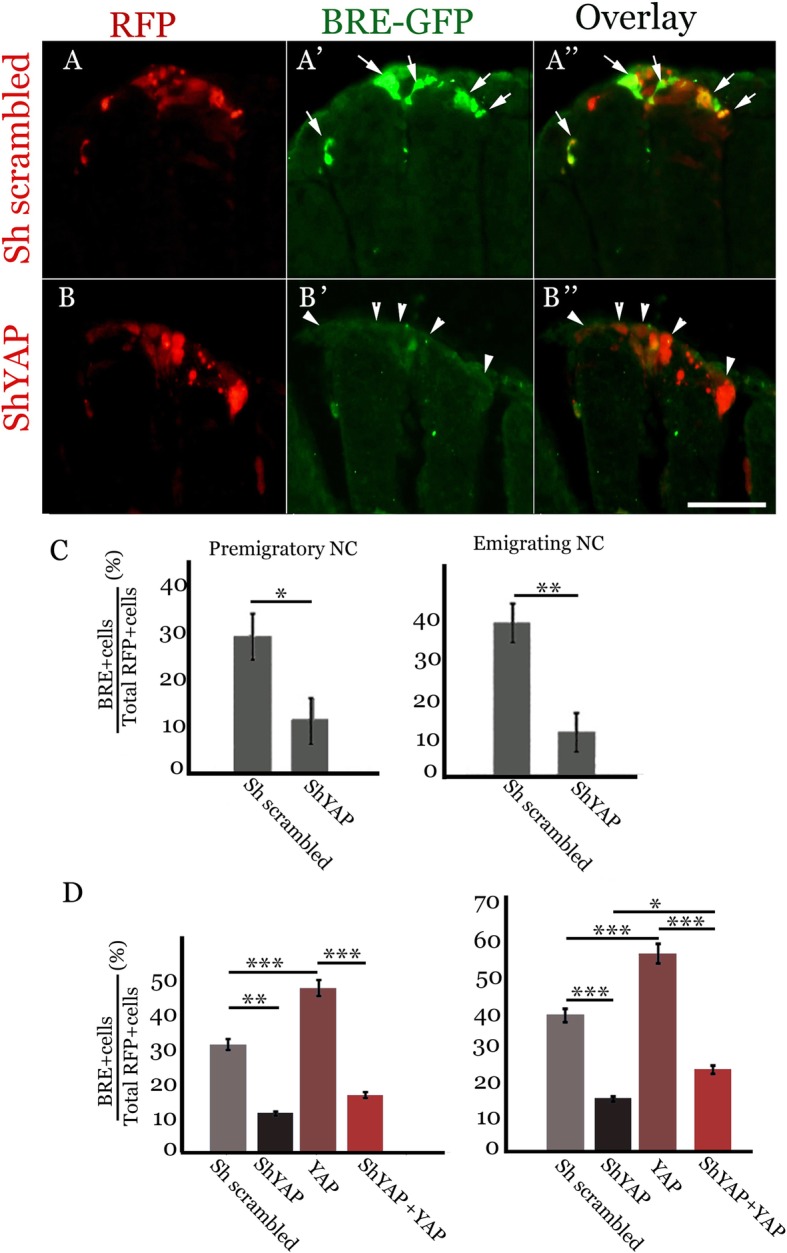
YAP signaling is required for BMP activity. Transverse sections of embryos co-electroporated with the BMP reporter BRE-GFP (green) and either a control scrambled RNA (**a**-**a**”), or shYAP (**b**-**b**″). Note the presence of double positive cells in controls (arrows in **a**”) and of mostly shYAP+/BRE-negative progenitors in the dorsal NT of shYAP-treated embryos (arrowheads in **b**″). (**c**) Left and right graphs show quantification of the percentage of double labeled progenitors out of total RFP+ cells in premigratory (*p < 0.001) and emigrating (**p < 0.0005) NC cells, respectively. (**d**) Left and right graphs show quantification of the percentage of double labeled progenitors out of total RFP+ cells in premigratory and emigrating NC cells, respectively. (*p < 0.004, **p < 0.0001, ***p < 0.00001). Bar = 50 μM

We previously found that BMP regulates NC EMT in the trunk of avian embryos by acting via Wnt signaling which in turn stimulates G1/S transition of the premigratory progenitors (see Introduction). Since YAP also stimulates G1/S transition, mitosis and NC emigration, we examined possible interactions between these pathways. First, scrambled RNA or shYAP were co-electroporated along with a genetic reporter for BMP activity, consisting of a BMP responsive element (BRE) driving expression of GFP [48–50]. Focal electroporations were directed to the dorsal NT at 15-18ss and embryos further incubated for 24 h. Double-labeled cells were observed in control embryos in both premigratory and emigrating NC (Fig. 6, A-A”,arrows, and C). In contrast, in the experimental embryos, a 2.6 and 3.4-fold decrease in the percentage of cells co-expressing BRE and shYAP was monitored, respectively, (arrowheads for shYAP+/BRE-negative cells in Fig.6b-b”, *N* = 8 for each treatment, *p* < 0.001 and *p* < 0.0005 for premigratory and emigrating cells, respectively). These results show that YAP is necessary for BMP activity in the dorsal NT.

To examine whether YAP is also sufficient for BMP activity, we overexpressed a plasmid encoding YAP and monitored the extent of BMP signaling in both premigratory and emigrating NC cells, respectively (Fig. 6d). Five embryos were analysed for each treatment. YAP overexpression caused a significant increase in the percentage of BRE-expressing cells in both domains when compared to controls (*p* < 0.00001), showing it is sufficient for stimulation of BMP activity in the NC. We further confirmed in this independent experiment that shYAP reduced the proportion of BRE-expressing cells (*p* < 0.0001 and *p* < 0.00001 for premigratory vs. emigrating cells, respectively) (Fig. 6d). When neural tubes were co-electroporated with both shYAP and YAP-encoding plasmids, no significant rescue vis-à-vis shYAP was observed in premigratory cells and a mild but significant increase was monitored in the emigrating progenitors (*p* < 0.004). Importantly, the percentage of cells exhibiting BMP activity in the combined treatment remained significantly lower when compared to YAP alone in both domains (*p* < 0.00001). Since the Yap we applied is a chick construct, it is expected that shYAP (also designed to a target chick sequence) abrogates both the endogenous YAP as well as the transfected one, at least partially. Hence, the present results confirm and further extend previous data showing the specificity and potency of the plasmids implemented [46].

**Fig. 7:**
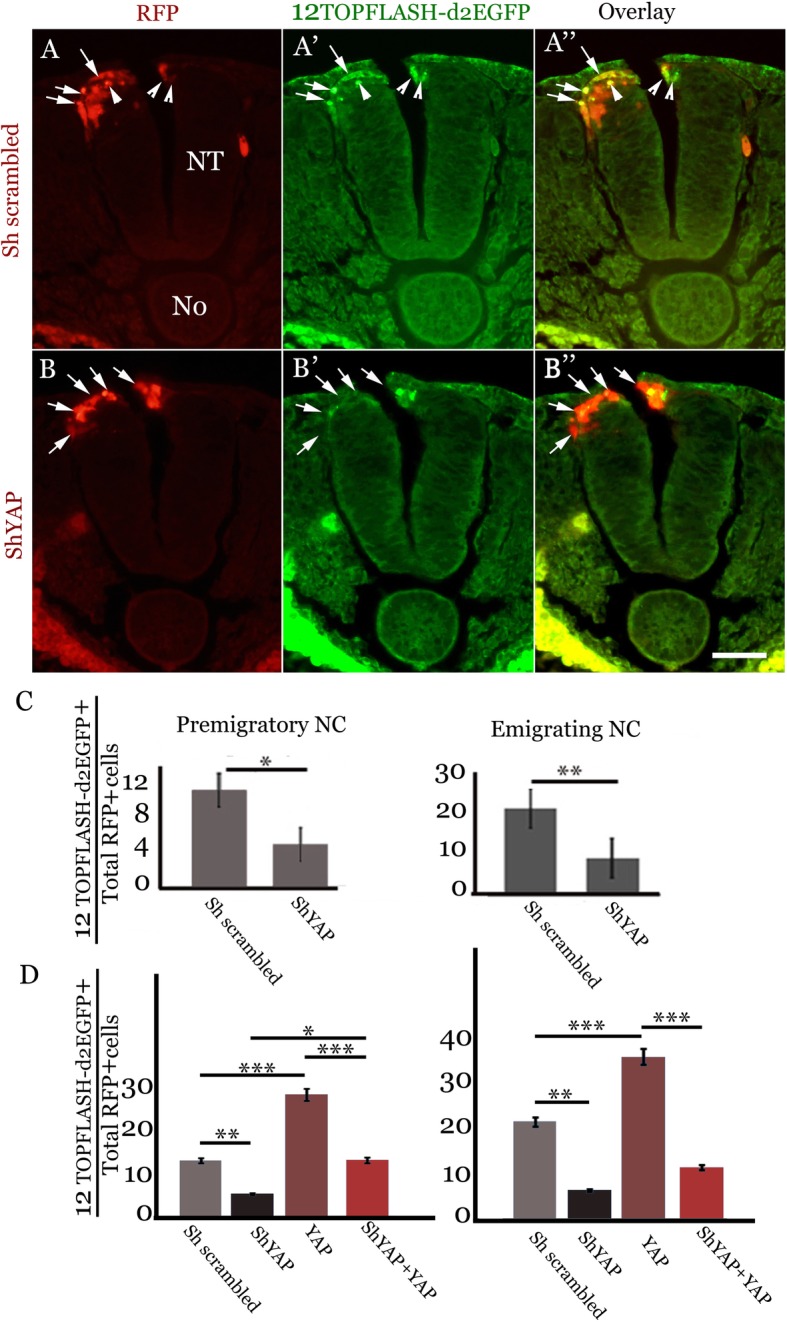
YAP is necessary for activity of the canonical Wnt pathway. Transverse sections of embryos co-electroporated with the 12TOPFLASH-d2EGFP reporter of canonical Wnt activity (green) and either a control scrambled RNA (**a**-**a**”), or shYAP (**b**-**b**″, red). Note the presence of double positive cells in controls (arrowheads in **a**’ and **a**” showing premigratory NC and arrows pointing to emigrating progenitors). In contrast, most shYAP+ progenitors are BRE-negative in the dorsal NT of shYAP-treated embryos (short arrows in **b**′,**b**″). (**c**) Left and right graphs show quantification of the percentage of double labeled progenitors out of total RFP+ cells in premigratory (*p < 0.001) and emigrating (**p < 0.0004) NC cells, respectively. (**d**) Left and right graphs show quantification of the percentage of double labeled progenitors out of total RFP+ cells in premigratory and emigrating NC cells, respectively. (*p < 0.03, **p < 0.0001, ***p < 0.00001). Bar = 50 μM

Next, we examined the effects of shYAP on β-catenin-dependent Wnt activity in the dorsal NT and emigrating NC cells. To this end, we implemented a reporter containing 12 TCF binding sites cloned upstream of a minimal promoter driving destabilized enhanced GFP (12 TOPFLASH-d2EGFP) [52]. In contrast to GFP in the BRE, the half-life of d2EGFP is only about two hours, hence, the signals observed reflect activity of the pathway in real time. Wnt activity was observed in both premigratory (arrowheads) and emigrating (arrows) progenitors under control conditions (Fig. 7, a-a”, c). Due to the lower stability of the d2EGFP compared to BRE-GFP, a lower percentage of double-positive progenitors was evident when compared to BRE (Figs. 6c and 7c). Importantly, shYAP further reduced the percentage of cells co-expressing shYAP and Wnt reporter in both premigratory and emigrating cells (arrows in Fig. 7, B-B″, C, *N* = 10 for each treatment, *p* < 0.001 and *p* < 0.0004, respectively). Thus, similar to BMP signaling activity, canonical Wnt activity is modulated by YAP signaling in both the dorsal NT and emigrating NC.

To examine whether YAP is also sufficient for Wnt activity, we overexpressed YAP and monitored the extent of Wnt signaling similar to what was performed for BMP in Fig.6d. Six embryos were counted for each treatment. YAP overexpression caused a significant increase in the percentage of cells exhibiting Wnt activity in both domains when compared to controls (*p* < 0.00001, Fig. 7d), corroborating that, like for BMP, YAP is sufficient for stimulation of Wnt activity in the NC. In contrast, shYAP decreased the proportion of cells expressing the Wnt reporter compared to controls (*p* < 0.0001 for both domains) (Fig. 7d and see also panel C). When both shYAP and YAP-encoding plasmids were electroporated, no significant rescue vis-à-vis shYAP was observed in the emigrating cells whereas a small but significant increase was monitored in the premigratory precursors (*p* < 0.03). Furthermore, the percentage of cells exhibiting Wnt reporter activity in the combined treatment remained significantly lower when compared to YAP alone in both premigratory and emigrating sites (*p* < 0.00001, Fig.7d). This result further confirms that shYAP raised against a chick sequence specifically inhibits both endogenous YAP as well as (at least partially) the transfected chick YAP. Together, our results demonstrate that YAP is necessary and sufficient for the activity of both BMP and Wnt in the early NC.

### BMP and Wnt are necessary for YAP activity

**Fig. 8:**
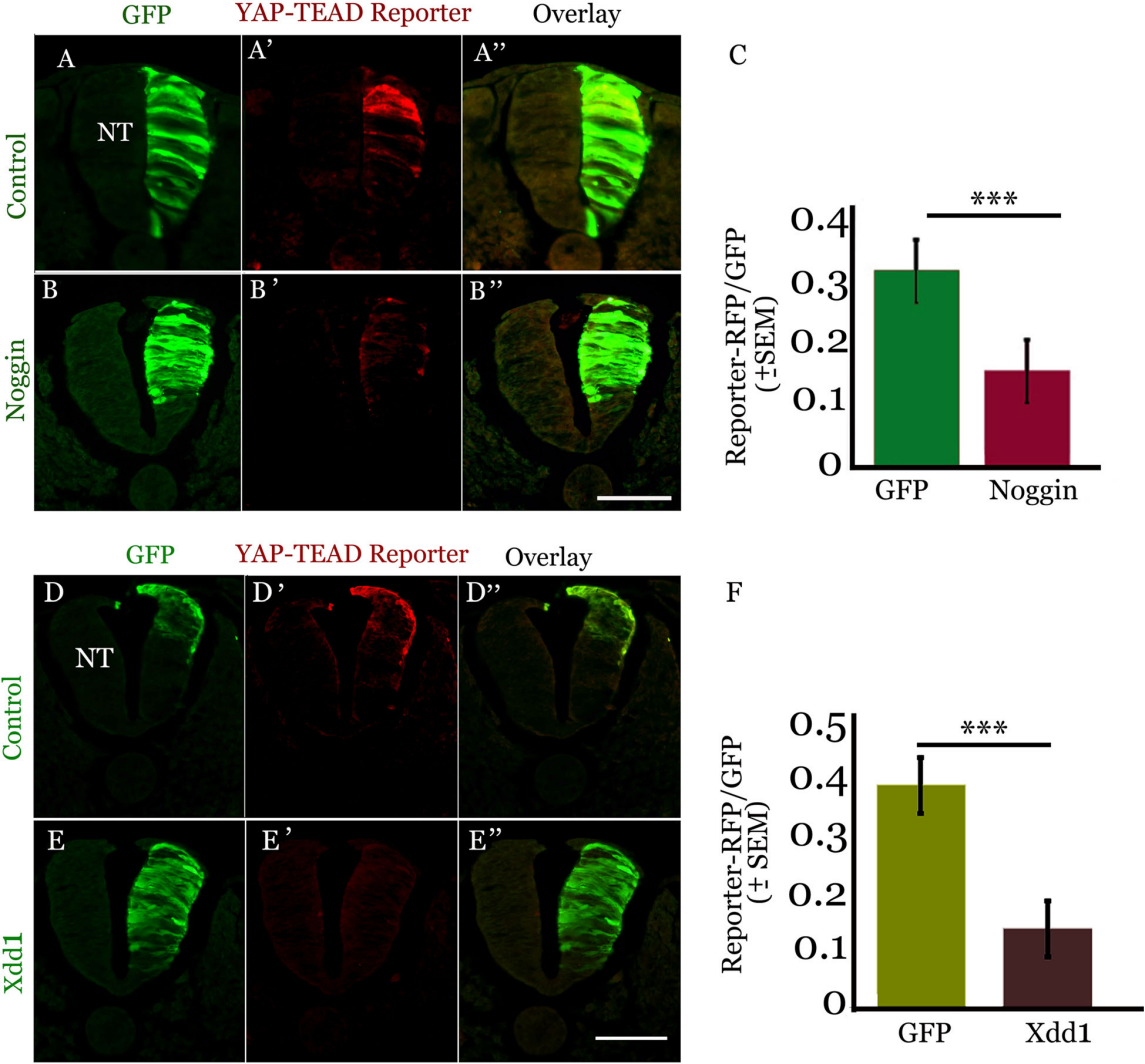
BMP and Wnt activities are needed for YAP signaling. (**a**-**b**″) Transverse sections of embryos co-electroporated with the YAP-TEAD reporter (red) and either control GFP (**a**-**a**”), or noggin (**b**-**b**″) (green). Note that inhibition of BMP by noggin reduced the intensity of reporter activity. (**c**) Quantification of reporter activity in the dorsal 20% of the neural tube (NT) (***p < 0.0001). (**d**-**e**”) Transverse sections of embryos co-electroporated with the YAP-TEAD reporter (red) and either control GFP (**d**-**d**””), or Xdd1 (**e**-**e**”) (green). Note that inhibition of Wnt by Xdd1 reduced the intensity of reporter activity. (**f**) Quantification of reporter activity in the dorsal 20% of the NT (***p < 0.0001). Bars = 50 μM

Since YAP modulates the activity of both BMP and of its downstream signal, Wnt, in the dorsal NT (Figs. 6 and 7), we next asked whether YAP activity is sensitive to changes in BMP and Wnt. First, hemi-NTs were co-electroporated with the BMP inhibitor Noggin and with the specific YAP-TEAD reporter (Fig.2). Twenty four hours following transfection, the YAP-TEAD reporter was apparent in the treated side of control embryos (Fig. 8, a-a”, c, *N* = 8). In contrast, its intensity was significantly reduced in Noggin-treated NTs (Fig. 8, b-b″, c, *N* = 6, *p* < 0.0001).

To examine whether Wnt signaling is also necessary for YAP activity, we overexpressed a truncated form of XDishevelled, a key component of the Wnt signaling pathway [62] harboring a partial deletion in the PDZ domain (Xdd1-GFP). Previous studies showed that Xdd1 acts in a dominant negative form to abolish both canonical as well as non-canonical Wnt signaling [63, 64], and inhibits NC EMT in avian embryos [18].

Hemi-NTs were co-electroporated with Xdd1 and with the YAP-TEAD reporter. A day later, Xdd1 significantly reduced the intensity of YAP activity when compared to controls (Fig. 8d-f, N = 6 for control and Xdd1, *p* < 0.0001).

Taken together, our results reveal a positive bidirectional interaction between BMP, WNT and YAP activities in the regulation of proliferation and emigration of NC progenitors.

### BMP and Wnt mediate YAP-dependent stimulation of NC EMT

We showed that YAP signaling is both necessary and sufficient for NC EMT (Fig.3). In addition, we documented that YAP is necessary for BMP and WNT activities and vice-versa (Figs. 6, 7 and 8). Since our previous findings demonstrated that both BMP and Wnt stimulate NC EMT (see Introduction), we examined a possible relationship between the above factors and YAP signaling in the regulation of NC emigration.

**Fig. 9:**
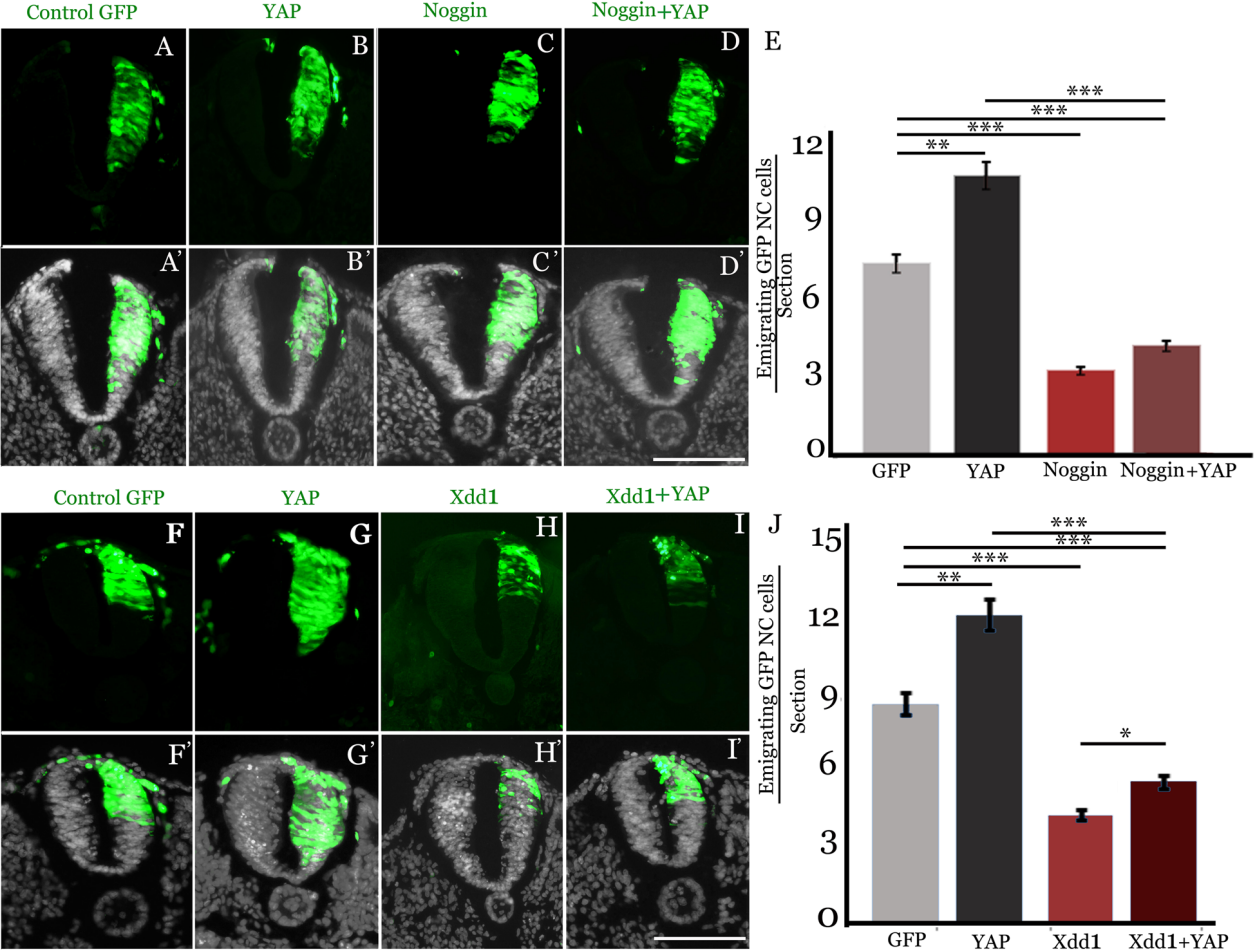
BMP and Wnt mediate YAP-dependent stimulation of NC EMT. (**a**-**d**’) Electroporation for 16 h of control GFP (**a**,**a**’), YAP (**b**,**b**′), Noggin (**c**,**c**′), or Noggin+YAP (**d**,**d**’). YAP stimulates NC EMT and Noggin inhibits the process. YAP is unable to rescue cell emigration in the absence of BMP signaling (Noggin+YAP). (**e**) Data quantification.**p < 0.001 for YAP/control; ***p < 0.0001 for Noggin/control and Noggin+YAP/YAP, and ***p < 0.0002 for Noggin+YAP/control. (**f**-**i**′) Electroporation for 16 h of control GFP (**f**,**f**′), YAP (**g**,**g**’), Xdd1 (**h**,**h**′), or Xdd1 + YAP (**i**,**i**′). YAP stimulates and Xdd1 inhibits NC EMT, respectively. YAP mildly rescues cell emigration in the absence of Wnt signaling when compared to Xdd1 alone (Xdd1 + YAP). (**e**) Data quantification. **p < 0.0006 for YAP/control; ***p < 0.0001 for Xdd1/control; ***p < 0.0003 for Xdd1 + YAP/control, ***p < 0.0001 for Xdd1 + YAP/YAP and *p < 0.04 for Xdd1 + YAP/Xdd1. (**a**’-**d**’ and **f**′-**i**′) Nuclei were stained with Hoechst (grey). Bars = 50 μM

As already shown (Fig. 3), overexpression of YAP significantly enhanced NC EMT when compared to control GFP (Fig. 9a-b’, e, N = 6 for both treatments, *p* < 0,001), and Noggin missexpression reduced NC EMT (Fig. 8c,c’, e, N = 6, *p* < 0.0001). Notably, co-treatment of Noggin and YAP (N = 6) failed to rescue NC EMT when compared to Noggin alone, and the number of emigrating cells remained significantly lower when compared to either control GFP (*p* < 0.0002) or YAP (*p* < 0.0001) (Fig. 8d,d’, e).

Next, we focused on the relationship between Wnt signaling and YAP in the context of NC EMT. We confirmed once more that overexpression of YAP significantly enhanced NC EMT when compared to a control plasmid (Fig. 9f-g”, j, *N* = 5 for both treatments, *p* < 0,0006), and Xdd1 missexpression inhibited NC EMT (Fig. 9h,h’, j, N = 5, *p* < 0.0001). Notably, co-treatment of Xdd1 and YAP (N = 5) only modestly rescued NC EMT when compared to Xdd1 alone (Fig.9i,i’, j, *p* < 0.004), yet NC emigration still remained significantly lower compared to either control GFP (*p* < 0.0003) or YAP (*p* < 0.0001).

Together, our results suggest that the stimulation of NC EMT by YAP depends on the integrity of both BMP and Wnt activities in the dorsal NT. Furthermore, the observation that YAP missexpression mildly rescued Xdd1-dependent inhibition of NC EMT is consistent with both Wnt as well as YAP acting at the level of NC proliferation; hence, relative amounts of either plasmid could alter the proliferative balance and consequently affect the extent of NC delamination. In contrast, BMP acts not only upstream of Wnt signaling in the present context but also of additional signaling systems controlling cell adhesion and the generation of motility, altogether involved in the regulation of NC EMT (see Introduction).

## Discussion

In this study, we demonstrate that YAP signaling plays a role in regulating the onset of NC EMT in the trunk of avian embryos. Moreover, our data provide a functional link between YAP and BMP signaling, a master regulator of NC EMT [17], and with canonical Wnt activity, previously shown to act downstream of BMP [18].

Both YAP immunoreactive protein and activity are evident in the dorsal NT harboring premigratory NC cells. Data from Xenopus tropicalis are consistent with our antibody results, and showed that, at the mRNA level, *YAP* is expressed in cranial premigratory and migrating cells [65]. Cao and colleagues documented expression of *YAP* mRNA in the chick embryo at a later stage (5–6 days), where transcripts were apparent throughout the ventricular zone of the spinal cord including the roof plate [46]. Also in the mouse, YAP protein was expressed in the NT including the premigratory NC where it colocalized with PAX3 [39]. Thus, our results documenting the presence of YAP in the premigratory domain of the NT confirm data from other species and further extend them to directly demonstrate that YAP is active in this domain.

With loss of YAP activity by YAP-NLS or shYAP, EMT and delamination of NC cells was significantly compromised. Conversely, more cells emigrated when YAP was overexpressed or the activity of the Hippo/MST2 kinase was abrogated. This demonstrates a pivotal effect of YAP on NC emigration *in ovo*. Likewise, using a morpholino approach in zebrafish, less crestin-positive migratory NC cells were observed in the cranial region of the morphant embryos [43]. In addition, a migratory NC phenotype was induced in a variety of human neural stem cell cultures subjected to siRNA-mediated knock-down of neurofibromatosis-2 (NF2), an upstream regulator of Hippo kinases and inhibitor of YAP activation [66].

In apparent contrast with the precedent studies, double-floxed *YAP* and *TAZ* mice, revealed no major defects in NC migration at embryonic days 9.5-E10.5 despite the lack of activation of Pax3 by the YAP/TAZ complex and development of a craniofacial phenotype [39]. Since this was based on a qualitative assessment, the possibility remains open that reduced and/or delayed NC emigration still occurred in the mutants. Alternatively, activation of Pax3 by YAP/TAZ, found by Manderfield et al. [39] to be TEAD-independent, may not be necessary for NC EMT. Other YAP targets, and/or binding to DNA via TEAD factors, might mediate the effects we monitored in avians. These apparent dissimilarities among species might also reflect a difference in the relative need for YAP signaling as a modulator of EMT that could be compensated for by additional signaling pathways.

Attenuation of YAP activity also affected G1/S transition and mitosis of the premigratory pool of NC progenitors, as well as, to a certain extent, also their survival. The latter might be directly affected by reduced YAP, or alternatively, be a secondary consequence of the failure of cells to proliferate and then delaminate from the NT. Regardless of the precise mechanism, the effects observed on cell cycle and apoptosis are in line with known effects of the Hippo/YAP pathway in other systems [29, 40, 46, 54]. Since G1/S transition and *cyclin D1* transcription in the premigratory NC are essential for NC EMT and are driven by BMP, Wnt and BMP-dependent proteolytic processing of N-cadherin [6, 12], it is possible that YAP primarily acts to stimulate BMP activity in the dorsal NT. In addition, YAP could directly activate specific cell cycle and EMT genes required for NC emigration, a question that remains to be addressed.

While this is the first study to document an interaction between YAP, BMP and Wnt pathways in NC cells, evidence from other systems suggests that these pathways regulate one another in a context dependent manner to properly regulate tissue growth, homeostasis and repair [67]. For example, BMP2/SMAD was shown to repress proliferation of murine embryonic neural stem cells through YAP [68]. In addition, Yap stabilizes SMAD1 to promote BMP2-induced neocortical astrocytic differentiation and vice-versa [69], a bidirectional interaction also observed in our system. In Drosphila, the homologues of BMP/YAP were reported to cooperate to promote tissue growth [70] and YAP was demonstrated to induce BMP in several cell types while also repressing canonical Wnt signaling by stimulating the production of Wnt inhibitors [71]. In another context, it was shown that Wnt-dependent signaling operates downstream of YAP to regulate formation and regeneration of the neuromuscular junction [72]. A mechanistic explanation for this interaction suggests that cytoplasmic YAP/TAZ are integral factors of the β-catenin destruction complex. Thus, cells devoid of Wnt signaling would sequester YAP/TAZ to the destruction complex and use them to degrade β-catenin. Wnt stimulation would then eliminate, or inhibit, cytoplasmic YAP/TAZ, causing β-catenin stabilization, as well as YAP/TAZ nuclear accumulation [73]. Furthermore, it was reported that β-catenin/TCF4 complexes bind a DNA enhancer element within the first intron of the YAP gene to drive YAP expression in colorectal carcinoma cells [74], thus highlighting a physical interaction at the DNA level. Additional studies reported on direct interactions between YAP, BMP and Wnt at the protein level through formation of active complexes. [75–79]. Together, evidence gathered from different biological systems supports the notion of direct interactions at both gene and protein levels between YAP, BMP and Wnt pathways, in further support of the present results. Future studies should address the precise molecular mechanism underlying the YAP/BMP/Wnt cross talk in premigratory NC.

Although the molecular details of the above interactions remain to be studied, their functional significance in EMT of NC cells has been clarified in the present work. In this regard, it is important to stress that besides its significance to embryonic development, EMT of the NC is an excellent model to investigate metastatic processes [80, 81]. Since YAP is an oncoprotein not only involved in normal development but also in driving tumor formation, EMT and metastasis [24, 82–84], elucidating its precise mechanism on NC EMT will be instrumental for understanding key events in oncogenesis.

## Conclusions

By implementing specific antibodies and a YAP/TAZ reporter, we first show that YAP is both expressed and is active in the dorsal NT that harbors premigratory NC. Furthermore, results of both gain and loss of YAP function in premigratory NC cells demonstrate that YAP activity is both necessary and sufficient to drive EMT of trunk level NC progenitors. This is associated with the regulation of G1/S transition, previously shown by us to be required for NC EMT that is triggered by BMP and by downstream acting Wnt. Additionally, NC survival is also reduced upon YAP attenuation, either as a direct consequence of compromised activity or as an indirect result of the failure of NC cells to proliferate or delaminate in a timely fashion. Finally, we show that YAP acts upstream of both BMP and canonical Wnt activities and that, in turn, BMP and Wnt activities regulate YAP, creating a positive bidirectional cross talk. Together, these results provide novel insights into the dynamic molecular network leading to emigration of NC cells from the central neuroepithelium.

## Data Availability

Not applicable.

## Abbreviations

BMP: Bone morphogenetic protein
DRG: Dorsal root ganglion
NC: Neural crest
NT: Neural tube
Xdd1: Xdishevelled
YAP: Yes associated protein
